# Predictive Mechanisms Are Not Involved the Same Way during Human-Human vs. Human-Machine Interactions: A Review

**DOI:** 10.3389/fnbot.2017.00052

**Published:** 2017-10-13

**Authors:** Aïsha Sahaï, Elisabeth Pacherie, Ouriel Grynszpan, Bruno Berberian

**Affiliations:** ^1^Département d'Etudes Cognitives, ENS, EHESS, Centre National de la Recherche Scientifique, Institut Jean-Nicod, PSL Research University, Paris, France; ^2^ONERA, The French Aerospace Lab, Département Traitement de l'Information et Systèmes, Salon-de-Provence, France; ^3^Institut des Systèmes Intelligents et de Robotique, Université Pierre et Marie Curie, Paris, France

**Keywords:** prediction, sense of agency, comparator model, joint action, HMI

## Abstract

Nowadays, interactions with others do not only involve human peers but also automated systems. Many studies suggest that the motor predictive systems that are engaged during action execution are also involved during joint actions with peers and during other human generated action observation. Indeed, the comparator model hypothesis suggests that the comparison between a predicted state and an estimated real state enables motor control, and by a similar functioning, understanding and anticipating observed actions. Such a mechanism allows making predictions about an ongoing action, and is essential to action regulation, especially during joint actions with peers. Interestingly, the same comparison process has been shown to be involved in the construction of an individual's sense of agency, both for self-generated and observed other human generated actions. However, the implication of such predictive mechanisms during interactions with machines is not consensual, probably due to the high heterogeneousness of the automata used in the experimentations, from very simplistic devices to full humanoid robots. The discrepancies that are observed during human/machine interactions could arise from the absence of action/observation matching abilities when interacting with traditional low-level automata. Consistently, the difficulties to build a joint agency with this kind of machines could stem from the same problem. In this context, we aim to review the studies investigating predictive mechanisms during social interactions with humans and with automated artificial systems. We will start by presenting human data that show the involvement of predictions in action control and in the sense of agency during social interactions. Thereafter, we will confront this literature with data from the robotic field. Finally, we will address the upcoming issues in the field of robotics related to automated systems aimed at acting as collaborative agents.

## Introduction

Human beings are intentional agents in the sense that their behaviors do not solely consist in reacting to external stimulations but also in deliberately acting to reshape their environment. However, many of our daily actions include conspecifics or even artificial automated systems. Cooperative tasks between humans and machines tend to gain ground as machines make tasks less demanding for the human operator and yield an increase in productivity (Kaber et al., 2000). Nevertheless, this can cost human mastery, particularly with difficulties in understanding and coordinating with artificial systems (Sarter and Woods, 1995; Billings, 1997; Sarter et al., 1997).

The inability to predict traditional artificial agents' behaviors has been proposed as a potential contributor of this phenomenon. Indeed, during social interactions with peers, humans can represent and predict their partner's action outcomes through the same sensorimotor system involved in action generation (Kilner et al., 2007). Evidence of an action/observation matching system was first discovered in the premotor area (or F5 area) of the monkey (Di Pellegrino et al., 1992; Rizzolatti and Arbib, 1998; Rizzolatti et al., 2001; Umiltà et al., 2001). It has been shown that the neurons of F5 area and the parietal cortex (the so-called “mirror neurons”) of the monkey could fire both when executing a goal-directed action and when observing the very same action performed by another individual (Keysers et al., 2003; Fogassi et al., 2005). Later, an analogous system (called “mirror system”)—mainly composed by the superior temporal sulcus and fronto-parietal connections—has been revealed in humans (Rizzolatti and Craighero, 2004; Keysers and Gazzola, 2009). It has been hypothesized that before action execution (self-generated or observed other-generated), the mirror system could simulate the motor command allowing the simulation content to be used to predict the consequences of the action, enhancing action control or implicit action understanding (Pacherie and Dokic, 2006). Specifically, it has been proposed that this kind of motor simulation supports our understanding of the low-level motor intentions of others (i.e., what type of action one is doing; (Rizzolatti et al., 2001), for a review see Rizzolatti and Sinigaglia, 2010) and also our understanding of others' higher-level prior intentions (i.e., why one is doing this action; Iacoboni et al., 2005). Note should be taken some authors argued simulating an agent's action through the mirror system was enough to understand motor intentions but not sufficient for prior intention understanding (Jacob and Jeannerod, 2005). Still, prediction plays a fundamental role in understanding observed actions performed by conspecifics and also in action coordination during joint actions with them.

Besides, there is evidence predictive mechanisms are not solely involved in action generation and understanding but also in the sense of self-agency. The sense of agency can be defined as the pre-reflexive experience of being in control of a voluntary performed action. Individuals' sense of agency can be estimated with explicit self-reported measures using Likert's scales or percentages (Sato and Yasuda, 2005; van der Wel et al., 2012; Chambon et al., 2013), or, in order to avoid compliance biases, with implicit measures, such as sensory attenuation (Weiss et al., 2011) or intentional binding (IB) (Haggard et al., 2002; for a review see Moore and Obhi, 2012). IB is the most common method in the experimental field and refers to the perceptive temporal attraction between the onset of a generated action (e.g., a key press) and the onset of its sensory consequence (e.g., a tone) that occurs when the action has been intentionally triggered. Several recent studies have supported that the sense of agency was not self-specific and that it could occur for other human generated actions during passive observation and joint action (Wohlschläger et al., 2003a,b; Poonian and Cunnington, 2013).

By contrast, the existence of deep discrepancies between the machine's real state and the operator's perception of its state is well documented (Norman, 1988). In particular, while humans can represent the actions of human partners and thus have successful human interactions, they experience difficulties in mirroring and fitting with typical automaton-generated actions (Wohlschläger et al., 2003a,b; Obhi and Hall, 2011b; Kuz et al., 2015). Moreover, some studies seem to indicate humans are impaired in accurately predicting and building a joint agency (i.e., a mutually interdependent control of the environment) when acting together with a machine (Glasauer et al., 2010; Obhi and Hall, 2011b). Interestingly, this trend fades when human-like properties are endorsed by the automata (Wohlschläger et al., 2003b; Glasauer et al., 2010). Turning traditional automated systems into collaborative agents is therefore one of the major challenges of the robotic sciences.

In this article, we sought to review the existing data on social interactions with humans and with artificial automated systems to better understand the problems' nature encountered during interactions with traditional automata, and finally to present some means to remedy these problems.

## Individual action

### Predictive systems for individual action generation

**Figure 1:**
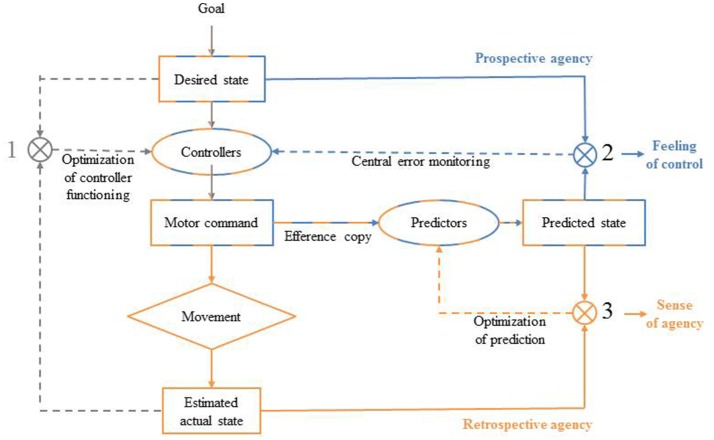
The comparator model (CM) from Frith et al. (2000).

Before one performs a deliberate goal-directed action, many sensorimotor information needs to be processed. Action control models suggest predictive mechanisms come into play during motor planning (see Figure 1; Frith et al., 2000; Blakemore et al., 2002; Synofzik et al., 2008). It has been proposed that the sensory consequences of a given action as soon as the motor command has been computed, before the onset of the action (Frith et al., 2000). According to the Comparator Model (CM), when an agent wants to achieve a goal, he or she will first represent a desired state (Frith et al., 2000). To meet this desired state, the agent has to program a specific motor command using sensory information about its own current state and the current state of the environment (e.g., affordances). The motor command is thus computed by an inverse model or set of paired controllers, which specifies the spatio-temporal dynamic of the appropriate action. To do so, controllers encode the dynamic properties of a given action each time this latter is performed in a specific sensori-motor context so they can then compute a motor command designed to yield the desired state given the current internal and external contexts. Once execution has started and feedback becomes available, the actual system's state is estimated and compared to the desired state, allowing error signals to be used to correct motor commands and to improve the functioning of the controllers (*comparison 1*, Figure 1). Before the motor command triggers the action, a copy of the motor command, called an efference copy, is made and sent to a forward model or a set of predictors that will compute the predicted consequences of the action. Then, this up-coming predicted state is compared to the initial desired state and in case of mismatch, an error signal is produced in advance of any sensory reafference (*comparison 2*, Figure 1). Finally, the estimated actual state can be compared to the predicted state and this comparison allows the set of predictors functioning to be optimized (*comparison 3*, Figure 1) (Frith et al., 2000). Such mechanisms are essential to anticipate and adjust our motor performances on-line, especially when the environment is uncertain and fluctuating. Importantly, in addition to their important role in the control of one's actions, there are reasons to think that predictive mechanisms are also involved in the sense of self-agency construction.

### Predictive systems for the sense of self-agency of individual actions

The sense of self-agency refers to the pre-reflexive experience of being in control of voluntary performed actions (Gallagher, 2000; Pacherie, 2007). Strong links have been made between the motor internal comparison processes and the sense of self-agency (Frith et al., 2000; Gallagher, 2000; Pacherie, 2007). The claim is that the quality of the match between a) the predicted state generated by predictors from the efference copy and the estimated real state generated by the real movement (Figure 1, *comparison 3*) and between b) the desired state and the predicted state (Figure 1, *comparison 2*) underlie the sense of self-agency (Frith, 2005; Sato and Yasuda, 2005; David et al., 2008; Moore and Fletcher, 2012). Specifically, *comparison 3* has been proposed to be mainly involved in self-agency attribution: sensory feedback that is congruent with the internal prediction will be self-attributed, while incongruent feedback will be attributed to external sources. The more incongruent the two signals are, the less sense of agency will the agent experience. For example, individuals misattribute self-generated sensory effects to an external source when an unexpected longer temporal delay follows their action (Sato and Yasuda, 2005). Additionally, Frith (2005) proposed that *comparison 2* was involved in the feeling of being in control of a given action because it allows for on-line adjustments of our own actions (Frith, 2005). Thus, the signals generated by both comparisons would contribute to an individual's sense of self-agency. Note that we acknowledge some limitations to the CM to explain by itself individual's sense of self-agency shaping (Synofzik et al., 2008; Desantis et al., 2012a; Frith, 2012). Indeed, the sense of agency may result from the dynamic integration of multifactorial sensorimotor cues (feed-forward cues, proprioception and sensory feedback) and circumstantial cues (intentions, thoughts, social and contextual cues) (Synofzik et al., 2008; Moore and Fletcher, 2012). For example, some authors have shown that when participants were induced the false thought that they were not the initiator of a tone but that a confederate presented as a genuine participant was, they experience less sense of agency (manifested by less IB when they were asked to judge the onset of the tone or less sensory attenuation when they were asked to judge the loudness of the tone) compared to when they thought that they were effectively the initiator of the sensory effect, even if in both cases it was the participant who had triggered the tone (Desantis et al., 2011, 2012b). However, this does not discredit the validity of the CM and we assume that sensorimotor predictive processes play a key role in the sense of self-agency of individual actions. Given that sensory feedback is needed in the CM (implying the action has been done), it has been considered as a retrospective process by several authors (Farrer and Frith, 2002; Synofzik et al., 2008; Chambon et al., 2013, 2014). Yet, in line with Synofzik et al. (2013), we consider that the CM is essentially a prospective process for the sense of agency because the predictive mechanism that occurs before action onset (i.e., the predictions about the sensory consequences of the action made through the efference copy) plays a crucial role in the model (Synofzik et al., 2013). Despite these divergent views, predictive mechanisms are still considered as a key component of the sense of agency.

**Figure 2:**
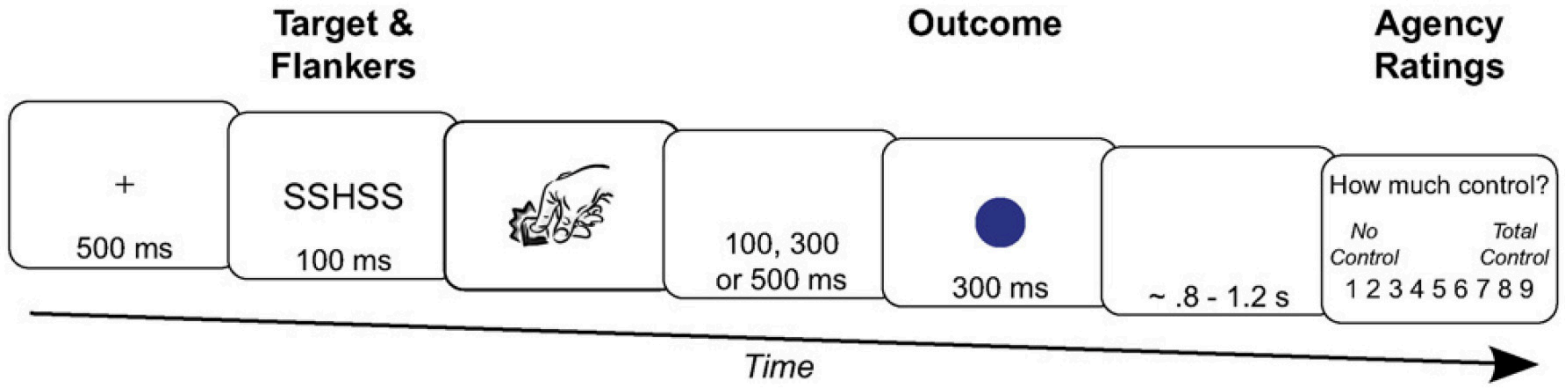
Timeline of an incongruent trial in the study of Sidarus and Haggard (2016). After the fixation cross apparition, the participants were primed with incongruent target (H) and flankers (S). Then, they had to press the key associated with the central target (e.g., the right key for the H target). The key press triggered an outcome color after a variable delay of 100, 300, or 500 ms. Finally, the participants had to rate with a scale their perceived control over the outcome apparition.

Moreover, the effect of action selection on human sense of agency has also been considered as an argument to consider the sense of agency as a prospective process (Chambon et al., 2013, 2014). Indeed, when action selection is easy, the sense of self-agency becomes stronger compared to conflictual action selection and as a consequence, it prospectively informs the sense of agency (Chambon et al., 2014; Sidarus and Haggard, 2016). For example, using an Eriksen flanker task, Sidarus and Haggard (2016) showed that participants' judgments of control were higher on congruent trials compared to neutral and incongruent ones (Sidarus and Haggard, 2016). The participants were presented a central target letter (S or H) surrounded by four non-target letters (two on the left and two on the right). Participants had to press a left key when the central target letter was an “S” and a right key when the central target was an “H.” The flanked letters could be congruent or incongruent with the central target. The detailed procedure for one trial is described on Figure 2. Because they were similar to the target, congruent distractors were meant to facilitate action selection whereas incongruent distractors were meant to induce a conflict in action selection as they were associated with the alternative action. Each key press triggered a specific sensory consequence. Participants had to report their feeling of control over the apparition of the sensory consequence on a Likert scale. Participants' reaction times were faster on congruent trials compared to incongruent trials. This facilitation effect during congruent trials went with higher explicit judgment of control compared to incongruent trials, thus demonstrating that changes in the flow of an ongoing action can modify an individual's sense of agency. Similarly, consistent priming about the sensory effects as well as about the ongoing action enhances participants' sense of self-agency (Wegner et al., 2004; Moore et al., 2009; Sidarus and Haggard, 2016).

Interestingly, such predictive mechanisms seem to be involved also in social interactions with human peers.

## Human-human interactions

### Predictive systems for shared action with other humans

Many studies suggest that acting jointly with someone else or observing someone else performing an action will engage the same representational structures underlying action execution. Joint actions can be defined as interdependent interactions involving two (or more) co-agents whose actions are directed at a common goal. Successful joint action requires co-agents accurately predicting consequences not only of their own actions but also of their co-agents' actions in order to correctly perform the complementary action in time and in space with respect to the joint goal. Based on one's own internal predictive models, motor simulation enables predictions about the actions of partners (Wolpert et al., 2003). Interestingly, several studies have shown that being involved in a joint action is not a necessary condition for predictive mechanisms activation, as these mechanisms can be elicited during the mere passive observation of others (Manera et al., 2011; Elsner et al., 2012, 2013). The claim that the same predictive mechanisms are involved during self-generated and observed other-generated actions is debated (Schilbach et al., 2013; Ruff and Fehr, 2014).

#### Joint actions with other humans

**Figure 3:**
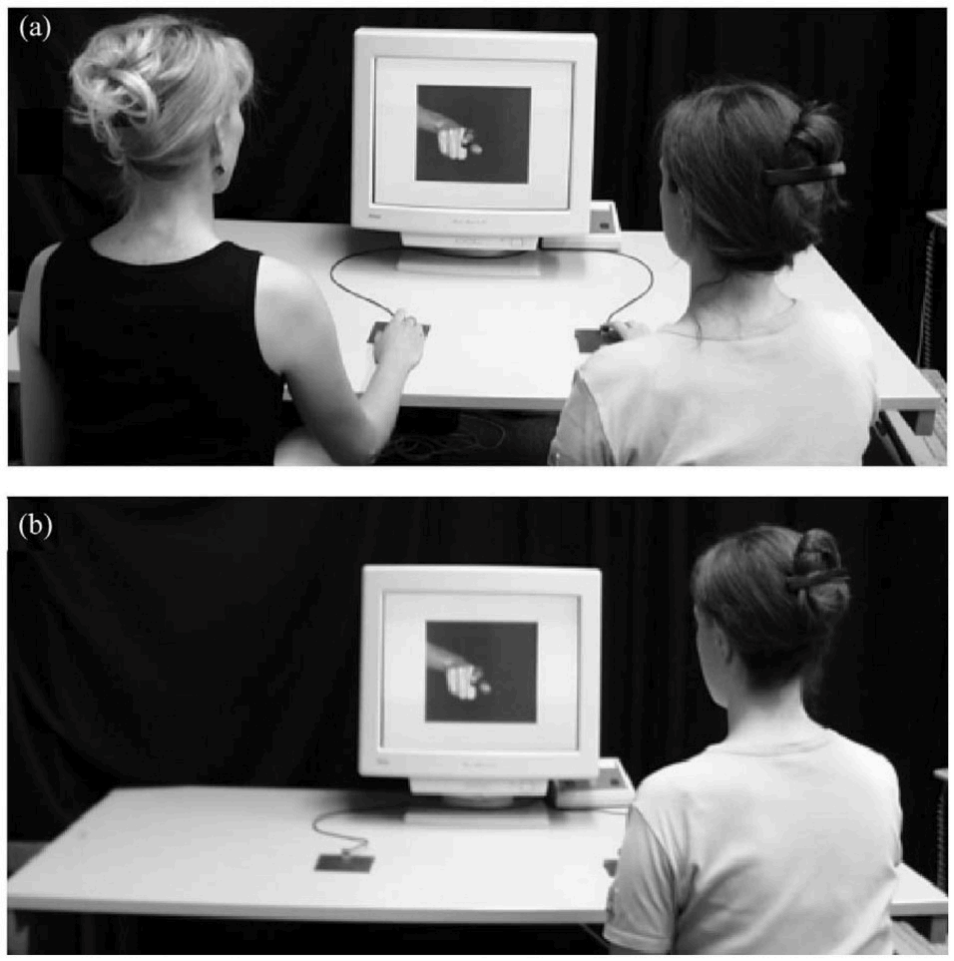
Experimental setting of the Social Simon task in the study of Sebanz et al. (2003). In the joint *go-nogo* condition **(a)**, two participants were sitting side by side and the target detection task was shared among then two individuals. In the individual *go-nogo* condition **(b)**, the participants were sitting alone and they had to perform the target detection on their own.

Using a joint Simon task, Sebanz et al. (2003) have found evidence of a social Simon effect, suggesting that during joint actions, actions of others are represented in our own motor plan (Sebanz et al., 2003). The standard Simon effect refers to the interference effect occuring when an individual has to respond with the right or left hand to a stimulus presented in an incongruent mapping location compared to a congruent mapping location (Simon and Small, 1969). A conflict occurs because two actions representations (i.e., the correct action to perform and the spatially-induced automatic activated action) are activated and the participant has to solve the conflict in order to select the accurate behavior. In Sebanz et al. (2003)'s first experiment, participants were shown pictures of a hand presented in the middle of the picture and pointing either to the left, to the middle or to the right side of the screen. On the index finger, there was a ring that could be either red or green. In the *two-choice* condition, the participant had to click on one of two boutons (left or right) according to the color of the ring. In the *joint go-nogo* condition, participants performed the task with a partner. Each of them had to respond to only one color by clicking on only one button. In the *individual go-nogo* condition, the participant was alone, and had to click only on one button associated with a given color. The experimental setting of the *go-nogo* conditions is presented in Figure 3. For all the conditions, the pointing direction of the finger was irrelevant for the task but could still automatically pre-activate the hand of the participant if the finger was pointing at this hand and thus facilitate the response (or create a conflict). Critically, in the *joint go-nogo* condition, each paired participants had to be responsive to only one attributed color so that they only had to perform one action type as for the *individual go-nogo* condition (left or right key press) and unlike the *two-choice* condition where each participant had to manage two colors so that they could perform two alternative actions (left *and* right key presses). The sole difference between the two go-nogo conditions was that in the *joint go-nogo* condition, another person was performing a complementary action beside the participant whereas in the *individual go-nogo* condition the participant was performing his or her task alone. The underlying idea was that if performance in the *joint go-nogo* condition were similar to performance in the *two-choice* condition, then it must involve similar inner representation of action (i.e., the two actions (left and right key presses) were both represented in the two conditions). Participants' reaction times were shorter when the index finger was pointing toward the correct button to push than when the index finger was pointing toward the opposite button to push, both during the *joint go-nogo* and the *two-choice* conditions. This finding supports the idea that during joint actions, partner's actions are functionally represented in our own motor system and activated in an automatic way, as they can interfere with our own performance, even when it is not task-relevant.

This result has been replicated by Sahaï et al. (2017) using a variant of the paradigm. In the *two-choice* condition, participants had to press a right key when a green circle appeared either on the left or right side of the screen, and a left key when a red circle appeared on the left or right side of the screen. In the *individual go-nogo* condition, the participants had to only detect the green circle that appeared either on the left or right side of the screen by pressing the right key. In the *joint go-nogo* human-human condition, participants sat the right side of the screen and a confederate the left side. Participants had to detect the green circles with the right key, and the confederate was to detect the red circles with the left key. The authors analyzed the reaction times of the participants. Consistently, the authors found a compatibility effect (faster reaction times when the target appeared on the same side as the participant response key compared to when the target appeared on the opposite side) only in the *two-choice* and *joint go-nogo human-human* conditions, thus supporting a social Simon effect.

**Figure 4:**
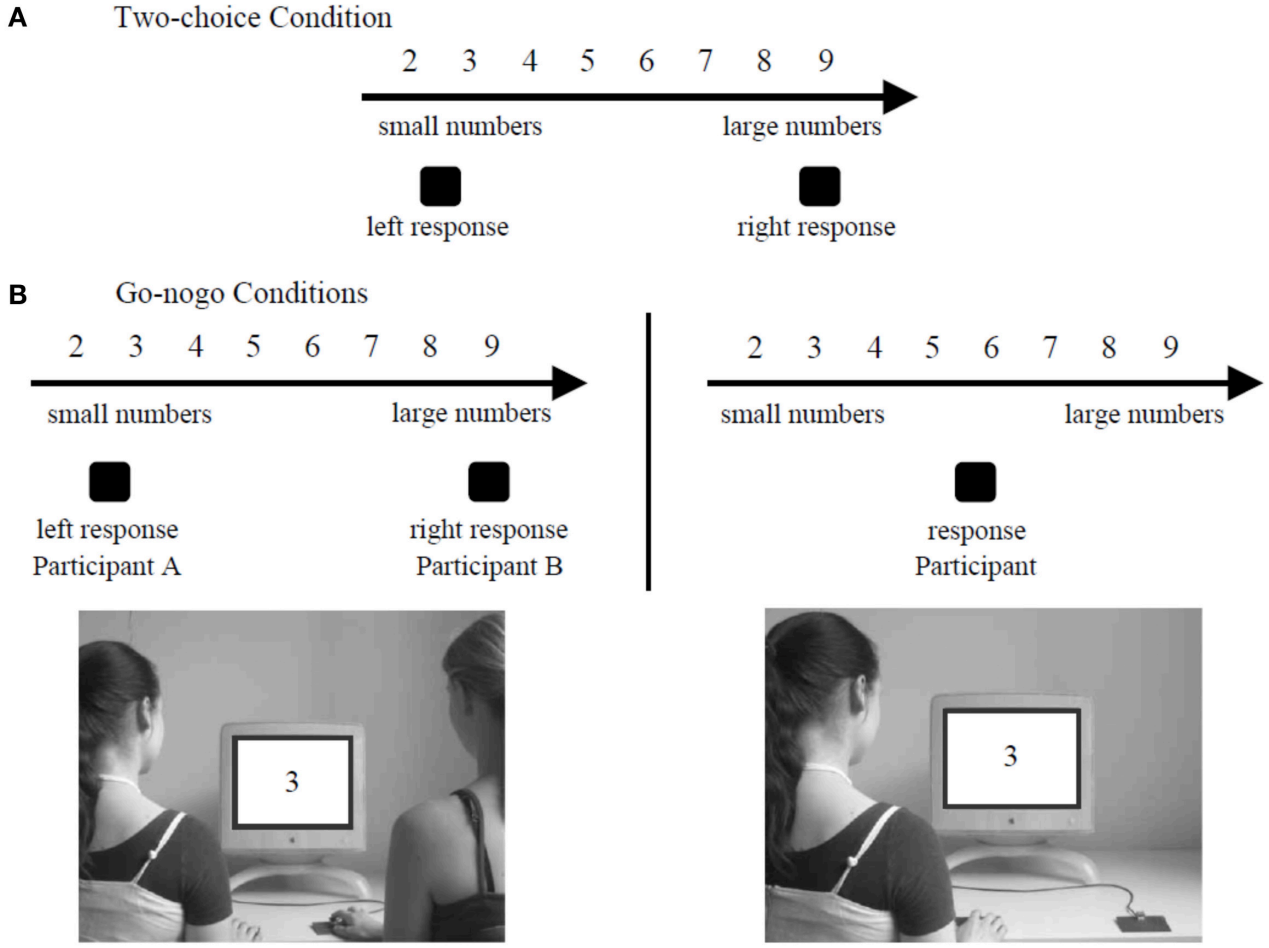
Experimental setting of the Social SNARC Simon task in the study of Atmaca et al. (2008). In the two-choice condition **(A)**, the participants had to detect both the even numbers with a button and the odd numbers with the opposite button. In the go-nogo conditions **(B)**, the participants had to detect only one sort of number (e.g. only the odd numbers) with one specific button.

The ability to represent the actions of a partner has also been shown with a more abstract concept, such as the SNARC effect (Atmaca et al., 2008). This effect relies on the fact that we represent numbers in our mind across a line where the small numbers are represented toward the beginning of the line (on the left) and the large numbers toward the other end of the line (Dehaene et al., 1993; Nuerk et al., 2005). It has been shown that participants were faster to respond to small numbers when they had to press a left key to answer and faster to respond to large numbers when they had to press a right key to answer (Dehaene et al., 1993). This occurs because the activation of the left/right part of the mental line triggers a pre-activation of left-/right- handed actions, leading to a facilitation effect. Atmaca et al. (2008) tested the hypothesis of a joint SNARC effect. In their study, participants had to respond to number parity for numbers between 2 and 9 with a left or right key. In the *joint go-nogo* condition, participants worked in pair. One had to react to the even numbers with one button whereas the other had to react to the odd numbers with the opposite button. In the *individual go-nogo* condition, participants worked alone. They had to perform exactly the same task than in the *joint go-nogo* condition except that there was no partner to perform a complementary action. In the *two-choice* condition, participants worked alone and had to respond both to the even and odd numbers with the appropriate button. The experimental setting of the Social SNARC Simon experiment is presented in Figure 4. Consistent with Sebanz et al. (2003), data showed a SNARC effect occuring during the *two-choice* condition and the *joint go-nogo* condition but not during the *individual go-nogo* condition. The presence of a joint SNARC effect suggests that each co-agent represented both his or her own action and the other's action so that conflict between two possible action alternatives occurred, as in the two-choice condition.

These behavioral data are supported by neurophysiological investigations. The contingent negative variation (CNV) is a slow negative brain wave arising during the last stages of action planning and can be considered as an index of other's actions representation as the CNV is effector-independent (Leuthold et al., 2004). Complex motor tasks lead to higher CNV amplitude (Cui et al., 2000). Authors have shown that the amplitude of the CNV was stronger when participants prepared a joint unimanual action compared to the same unimanual action realized in a personal way (Kourtis et al., 2014). This pattern of results has been proposed to reflect that participants have implicitly represented the action of their partner in their own system during the joint action so that the content represented was more complex (explaining the CNV enhancement) whereas it was not the case during the individual action (Kourtis et al., 2014).

In sum, when we perform joint actions with other people, we automatically represent the partner's task in our own motor planning. Hence, through the same predictive mechanisms underliying action execution, it is possible to anticipate the motor goal of the partner's action with the aim to promote successful interactions.

#### Passive observation of other humans

Recent theories of action control suggest that internal models involved during action execution are also engaged during goal-directed action observation (Kilner et al., 2007; Picard and Friston, 2014). According to the predictive coding account, low-level visual information is translated into high-level representation of the goal underpinning the observed action through the superior temporal sulcus and fronto-central areas *(prediction 1)*. Then, based on this prior inference about the goal of the observed action, one can predict the motor command of the observed action *(prediction 2)*. Based on this motor command prediction, one can predict the kinematics of the observed person thanks to one's own motor system *(prediction 3)*. Finally, the comparison between the predicted kinematics and the actual observed kinematics will produce a prediction error improving the initial inferred representation of the other's motor command *(prediction 2)* yielding a more accurate motor command estimate *(prediction 4)*. Likewise, the comparison between the prior predicted motor command *(prediction 2)* and the new improved motor command estimate *(prediction 4)* would improve the inferred goal estimate. Thus, one can infer the causes of an observed action by minimizing the prediction errors at the intention, goal, motor and kinematic levels engaged during action observation (Kilner et al., 2007; Picard and Friston, 2014).

Supporting the predictive coding account, it has been shown we can discriminate other's tiny variations of kinematics from our own motor experience (Manera et al., 2011). Manera et al. (2011) presented to participants videos showing either an actor who would next cooperate or compete with another player (not shown) in order to build a tower with small blocks of wood, or who was performing the building task alone, or showing instead point light (in which each point light corresponds to a hand joint) reflecting these same actions. The videos were cut just after the actor had grabbed the wooden block to perform the action. The task of the participants was to categorize the prior intention of the actor (cooperation vs. competition). The results showed that participants succeeded in categorizing the different social intentions both in standard and point light conditions. It has been hypothesized that this ability relied on motor simulation during action observation (Manera et al., 2011). Indeed, as prior intentions are embodied in kinematics, by simulating observed other-generated actions, one can understand what the observed agent's intention with the help of one's own motor experience (Iacoboni et al., 2005; Georgiou et al., 2007). Prior expectation about an agent's intention can modulate observers' correct intention inference (Chambon et al., 2011). For example, when participants had to infer the intention of an observed actor (e.g., to cooperate or to compete), they could not disengage themselves from induced implicit prior thought even when the amount of visual information was sufficient to make relevant predictions.

Furthermore, it has been shown individuals can predict an action goal before the action ends, provided that the action conforms to biological motion rules (Elsner et al., 2012). In their study, participants were shown video clips of a point light display (in which each point light corresponds to a hand joint) moving toward a target object. The kinematic pattern of the action was either biological or non-biological. In the biological condition, participants shifted their gaze toward the target object before the end of the action whereas there was no anticipatory behavior in the non-biological condition. Furthermore, in another study, Elsner et al. (2013) induced a transcranial magnetic stimulation (TMS) over either the hand's motor cerebral area or the leg cerebral area of participants looking at movies of point light reaching-grasping actions. They found participants' predictive gaze behavior was slowed down when the motor hand area was stimulated but not when participants had the leg motor area stimulation or without TMS (i.e., in the control condition), arguing for a somatotopical recruitment of observers' motor system during action observation. Hence, during the passive observation of others, motion cues are used to anticipate others' action goals, in accordance with the predictive coding theory.

The involvement of our own motor system during action observation is also supported by work on expertise (Calvo-Merino et al., 2004; Aglioti et al., 2008). For exemple, Aglioti et al. (2008) showed free basketball shot movies to participants who had to predict whether the ball would go in or out the basket (or if they did not know) by pressing a specific key. There were three groups of participants: expert basketball players, expert watchers with a similar visual experience, such as coaches or sport journalists, and novice basketball players. The authors showed expert basketball players were better at predicting successful free shots before the actor had thrown the ball using kinematic cues, in comparison with expert watchers and novices. Furthermore, using TMS, they found the basketball shot movies induced an increase of the corticospinal excitability both in the expert player and watcher groups but not in the novice group. Moreover, the observation of the erroneous free shots induced a greater corticospinal excitability than observation of the successful free shots but only in the expert player group. This motor facilitation in the expert player group underlines the existence of a fine-tuned action/observation matching system established through motor practice (Aglioti et al., 2008). Finally, neuroimaging data have shown the activation of brain areas well-known to be involved in predictive mechanisms during other-generated actions observation was mainly influenced by self-acquired motor skills, such as ballet or capoeira movements (Calvo-Merino et al., 2004).

Unlike individual actions, joint actions imply that the agents make additional planning to take into consideration other's intentions. They predict the partner's state because her or his sensorimotor information is not available. In accordance with the predictive coding theory, behavioral and neuroimaging data suggest the actions of others are spontaneously mapped onto our own motor system during social interactions, allowing action understanding and prediction. This may be a predisposition selected in the course of evolution to promote easy and efficient social interactions with conspecifics.

### Predictive systems for the sense of we-agency with other humans

Prediction is also important for the experience of agency during our interactions with human peers. Indeed, it has been proposed that the cognitive mechanisms that are involved in the sense of agency during individual actions are of the same kind as those underliying the sense of agency during joint-action of other humans (Pacherie, 2012).

#### The sense of agency during joint actions with other humans

Numerous authors have tried to understand how the actions of others could affect one's own sense of control during joint actions (i.e., joint agency) (Obhi and Hall, 2011a,b; Dewey et al., 2014; Sahaï et al., 2017).

In Obhi and Hall (2011a)'s study, paired participants were asked to act jointly by pressing a spacebar at will. In the first experiment, whenever one person pressed the spacebar first, the other had to also press the spacebar as soon as possible. The first key press triggered a tone 200 ms later. In this setting, both participants co-intended to trigger the sensory consequence. In the second experiment, one participant was instructed to press a spacebar at the time of his or her choice while the other had to press the spacebar as soon as possible after the initiator's key press. In this context, the sensory consequence was triggered by a personal intention. In both experiments, participants were asked to report their feeling of causal responsibility (i.e., a subjective experience of agency) using a percentage scale and also to judge the onset time of the first key press or the onset of the tone. Interestingly, in both experiments, although only the initiator reported a reliable feeling of causal responsibility, both individuals demonstrated IB. This finding has been explained by the spontaneous and pre-reflexive “we identity” formation occuring when two humans cooperate (Searle, 1983; Crivelli and Balconi, 2010). This new agentive identity leads individuals to experience agency as soon as one of the two had performed a goal-directed action.

In a second study, Obhi and Hall (2011b) placed participants in a room with a confederate (presented as a genuine participant), separated by a curtain. In the *operant* condition, participants were asked to tap the touchpad at the time of their choice, which triggered a tone after 200 ms. In the *action alone* condition, participants were asked to tap the touchpad at will, but their action was not followed by a tone. In the *tone alone* condition, a tone was externally generated. Participants thought the confederate could also cause the tone if they had tapped the touchpad before them but in fact, it was always the naïve participant who was performing the action triggering the tone. After each trial, a randomized false feedback about the initiator's identity was given to the participants (self, other or indistinguishable). Participants had to judge the onset time of their own action or the onset time of the tone with the help of a clock and to explicitly report their belief about who had caused the tone. Data showed that when the participants' action triggered an effect, the onset time of the action was perceived later than it was when the action did not cause any effect. Similarly, the perceived onset time of the tone in the *operant* condition was perceived differently from the *tone alone* condition. Even when they were convinced the confederate's action had caused the tone, they always demonstrated IB that was indistinguishable from the binding they showed when they believed that their own action had caused the sensory effect. The authors interpreted this phenomenon in terms of “we identity”: when two human partners are involved in a task together, a “we” agentic identity would be automatically formed, leading individuals to feel a sense of agency for actions that have been triggered by their partner, even if visual information concerning the action of the other is missing. However, in Obhi and Hall (2011b)'s study it was always the naïve participant who caused the tone. Indeed, participants were induced the false belief that their partner could also trigger the sensory effect if they had tapped a touchpad before them. Hence, these findings can be explained in terms of the predictive model of action. Regardless of participants' thoughts, they always performed the key press so that motoric information was always available. In addition, participants had to judge the onset of their own action and not the supposed onset of the action of the partner. Thus, the IB effect found might reflect the sense of self-agency of the participant himself or herself, which arises from the match between the predicted state (through the forward model) and the estimate actual state.

Finally, Dewey et al. (2014, experiments 2 and 3) have shown that in a joint task where participants perform complementary actions, their judgments of control were not just based on the effect predictability of their own actions but is also influenced by the amount of control exhibited by the team as a whole. In the study, the authors asked paired participants to track a target moving on a horizontal axis on a screen with a joystick (each participant had her or his own joystick). Each participant could control only one specific direction of the tracker (to the right or to the left) so that the contributions of both agents were required to succeed on the task. On some trials, either one or both of the participants' joysticks were turned off and/or noisy perturbations were induced on the joystick motion. The participants' judgments of control where highest when both joysticks were activated and the noise was turned off. This could be interpreted either as evidence that in these joint tasks participants evaluate their control from the perspective of the team rather than from their own egocentric perspective (joint control hypothesis) or as evidence of a self-serving bias, where participants attribute more control to themselves when the action is successful. To adjudicate between these two possibilities, Dewey et al repeated the experiment, but asked participants to rate their own control on half of the trials and their partner's control on the other half of the trials. Results indicated that both self and other judgments were highest in the condition where the two joysticks were activated and the noise was off. This indicates that collaborative actions, where co-agents make complementary contributions and the action effects produced by the partner are predictable, contribute to a sense of “we-ness,” where participants experience a shared sense of control over the joint task (Dewey et al., 2014). In addition, recent investigations have shown that during joint actions where participants had asymmetric roles, being a leader or a follower did not modulate the individual's judgements of control provided that the final goal is equally shared and not imposed by the leader (van der Wel, 2015). These data support the idea that during joint actions, a we-mode is running so that individual actions are turned into common actions (Searle, 1983; Crivelli and Balconi, 2010). Indeed, in this particular context of joint actions, the sense of control does not rely solely on the specific contributions of each individual but rather on the group performance.

This pattern of results is also found when the joint task is more conceptual and does not involve a common object to act on. Indeed, Sahaï et al. (2017)'s added a temporal delay estimation task to the Simon task. Moreover, in addition to the *two-*choice, the *individual go-nogo* and human *joint go-nogo* conditions, there was a *human observation* condition in which the participants had to passively observe the confederate performing the Simon task. Each accurate target detection made by the confederate or by the participants triggered a tone after a random delay. The participants had to report orally the perceived delay between the onset of the detection and the onset of the tone. The authors found that the participants made shorter temporal estimations when they had to judge the confederate-generated action in the *human joint go-nogo* condition compared to when they had to judge the very same action performed in the *human observation* condition. This brings additional support to the hypothesis that being engaged in a social collaboration with a partner can enhance the observer's sense of agency compared to the mere passive observation.

The predictive coding account allows us to understand how individuals can have a sense of agency for actions that have been realized by a partner. Nonetheless, it seems that similar mechanisms operate during the mere passive observation of others, maybe in a lesser extent.

#### The sense of agency during passive observation of other humans

Even when no common goal is shared, an individual can have a sense of agency during the observation of other-generated actions. Indeed, the activation of the observer's predictive systems links the action of the other to the observer's own motor system leading to a sense of agency enhancement. Thus, several studies have found IB phenomena for observed actions initiated by another person than the participant when this participant was not involved in the production of the outcome at all (Wohlschläger et al., 2003a,b; Moore et al., 2013; Poonian and Cunnington, 2013; Pfister et al., 2014; Poonian et al., 2015).

For example, using an explicit judgment methodology, Wegner et al. (2004, experiment 1 and 2) have shown that participants reported a higher degree of vicarious sense of agency when they were consistently primed about the on-going observed action compared to when they were not (Wegner et al., 2004). In their experiment, the participants were paired with helpers. Both participants and helpers wore headphones. Hand helpers were told that they would hear a sequence of instructions on what hand actions to make over the headphones, while participants were told that they might or might not hear anything over their headphones and that whatever was heard might or might not relate to the actions of the helper. Then, the participants had to evaluate with a Likert scale their feeling of vicariant control over the helper's actions. The results showed that when the participants were correctly primed by headphone instructions about the helper's action, they rated vicarious control over the observed action higher than when they received prior incorrect information. Such a finding indicates that motor intention understanding—mainly achieved through the forward model—is engaged in the feeling of vicariant control.

In Wohlschläger et al. (2003b)'s experiment, participants were placed in front of a lever that triggered a tone 250 ms after being pushed on. There were two conditions involving humans. In the *self-action* condition, participants had to press the lever at the time of their own choice and in the *other-action* condition participants observed the experimenter press the lever at the time of his choice. In both conditions, participants were asked to estimate the onset time of the lever press. The results showed that the perceived onset time of the action in the *self-action* condition was perceived in a similar fashion as in the *other-action* condition. Hence, the participants made IB independently of the generator of the action. This result emphasized that self-generated actions and observed other-generated actions are experienced similarly. The authors argued that the conscious experience of action was shared out across agents through the neural network involved both in action execution and action understanding (Wohlschläger et al., 2003b). It is possible that the other-generated action was simulated in the observer's predictive system so that a high level of congruency between the predicted state derived from the efference copy and the estimated real state generated by the real movement observation occurred. Note should be taken that other studies using a similar paradigm failed to found binding effect during action observation (Engbert and Wohlschläger, 2007; Engbert et al., 2008). However, in Engbert et al. (2008)'s study, the authors have implemented three different delays between the onset of the action and the onset of the tone (200, 250, and 300 ms), making predictability less important compared to the study of Wohlschläger et al. (2003b) where there was only one delay. According to Engbert et al. (2008), efferent information plays a key role in individual's sense of agency and the IB for other-generated action is an artifact from high predictable delays.

Still, an ERP study conducted by Poonian et al. (2015) has supported the idea of a common processing of self-generated and observed other-generated actions. In their experiment, participants had to press a key at the moment of their own choice or to observe a video where someone else was doing the same action. The key presses triggered a tone after a certain delay. In the control condition, there was no key press but the tone was presented twice, each presentation separated by a certain delay. For all conditions, participants had to estimate the perceived temporal interval between the first and the second event. The electrical activity of the participants' brain was recorded all along. Data showed that participants displayed IB between their own action and the subsequent tone as well as the delay between the observed action and the subsequent tone. For the control condition (externally-triggered tones), the delay between the two tones was overestimated. In addition, authors also used electroencephalography to measure the N1 wave suppression which is a mechanism associated with sensory attenuation that is considered as another implicit indicator of the sense of agency. The amplitude of the N1 component was reduced during the perception of the self-generated tone to the same extent that during the perception of the other-generated tone. For the control condition, N1 suppression was enhanced during the perception of the second tone compared to the first one because the sensory effect had already been heard once. To conclude, N1 suppression does occur also when hearing a sound that has been generated by another observed human (Poonian et al., 2015). These results are supported by the study of Moore et al. (2013). Indeed, using fMRI the authors have shown that the hemodynamic response of the cerebral areas underlying the sense of self-agency was similar for self-generated actions and observed other-generated actions (Moore et al., 2013).

Investigations have also focused on the effects of social variables on the sense of agency (Pfister et al., 2014). In Pfister et al. (2014)'s study, participants were paired. One assumed a leader role and the other a follower role. The leader had to press a key at the moment of his or her own choice and the key press triggered a tone after a certain delay (interval 1). This tone served as a go-signal for the follower to press his or her own key (interval 2). The follower's key press could trigger a tone after a random delay (interval 3) or no tone at all. Both the leader and the follower had to verbally judge the interval lengths. The results showed that the leader's interval estimations were always shorter than the follower's interval estimations, meaning that the leaders always made more IB than the followers. In addition, as the initiator of the action, the leader made IB while the follower, as the observer, did not (interval 1). For the interval 2 estimation, the leader made IB for the follower's action but the follower did not. Hence, leaders' sense of agency does not only concern their own actions and their adjacent effect but also predictable actions of other agents. Finally, when the follower's key press generated a sensory consequence, the temporal interval estimations (interval 3) were similar for the leader and the follower: no participant made IB. That is, the follower never experienced a sense of agency neither over his or her own action and their effects nor over those of the leader.

These different studies emphasize the importance of the predictive mechanisms in human-human interaction. The ability to predict appears as a fundamental component for both understanding and coordinating with others, but also a core component in our feeling of control in joint action. Interesting issues stem from the growing role of human machine interaction: can we take benefits from our predictive mechanisms when interacting with automated artificial agent?

## Human-machine interactions

### Predictive systems for shared action with machines

**Figure 5:**
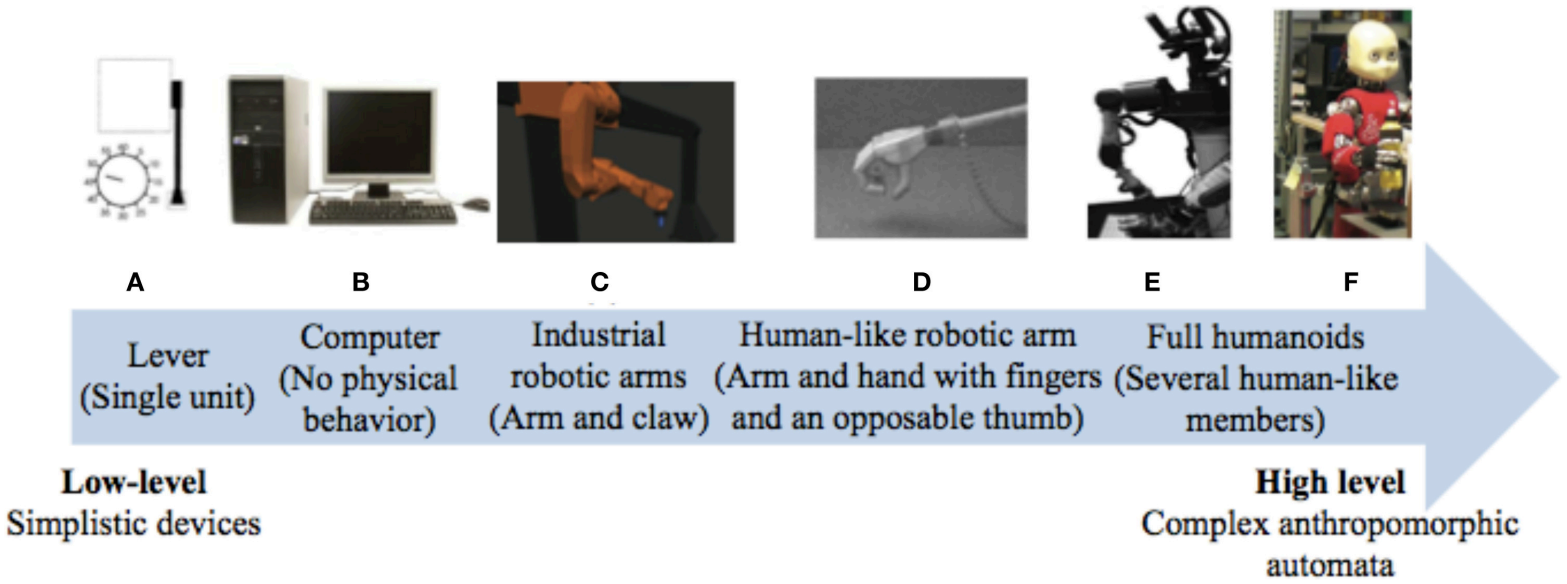
Varieties of artificial automated systems used in the research on the sense of agency and/or joint actions with robots. **(A)** A computer in Sahaï et al. (2017), **(B)** a lever in Wohlschläger et al. (2003b), **(C)** an industrial robotic arm in Kuz et al. (2015), **(D)** a human-like robotic arm in Oberman et al. (2007), **(E)** a humanoid with torso, eyes-cameras, arms, and fingers in Stenzel et al. (2012) and **(F)** a humanoid with torso, a human-like face, arms, and fingers in Sciutti et al. (2014).

Robotic research has been deeply interested in optimizing human–machine interactions in order to enhance efficiency and safety. So far, studies had involved a large variety of different automated systems with varying complexity, from computers to robotic arms, from levers to full humanoids (see Figure 5 for an overview). Yet, there has been a trend toward machine humanization during these recent years. While some researchers took an interest in humanizing the external appearance of the machine (Wohlschläger et al., 2003b; Riek et al., 2009), others have focused on its motor characteristics (Kajikawa and Ishikawa, 2000; Glasauer et al., 2010). Interestingly, current research tends to show that human-like machine-generated actions can engage the same predictive mechanisms that are brought into play during human interactions whereas this ability remain impaired during interactions with traditional automata.

#### Joint actions with machines

##### Joint actions with traditional automated systems

Co-represention of computer-generated actions in the human motor system during joint actions has been addressed by Sahaï et al. (2017). In their study, there was a *human-computer joint go-nogo* condition wherein participants had to act jointly with a computer in order to realize a target detection task. The participants had to detect one type of target (e.g., a green circle) that could appear on the left or the right of a screen with a right key while the computer was to detect an alternative target (e.g., a red circle). The author found no compatibility effect (i.e., no faster reaction times for the compatible trials compared to the incompatible trials). This absence of motor interference on the incongruent trials suggests that participants were not able to represent the computer-generated actions into their cognitive system.

It has also been shown that motor expertise in tennis played a crucial role during human interactions but had no influence during human-machine interactions. Indeed, expert tennis players have faster reaction times than novice tennis players when they are playing with another human but they do not benefit from their experience anymore when they are playing against a cloaked ball machine (Mann et al., 2007). Such results confirm the role of our own motor planning in our representation of the partner's action, and the difficulties that could arise in cases of non-human co-agent. Indeed, the comparator model implies that individuals make predictions about the ongoing other-generated action based on their own motor repertoire.

##### Joint actions with humanized automated systems

**Figure 6:**
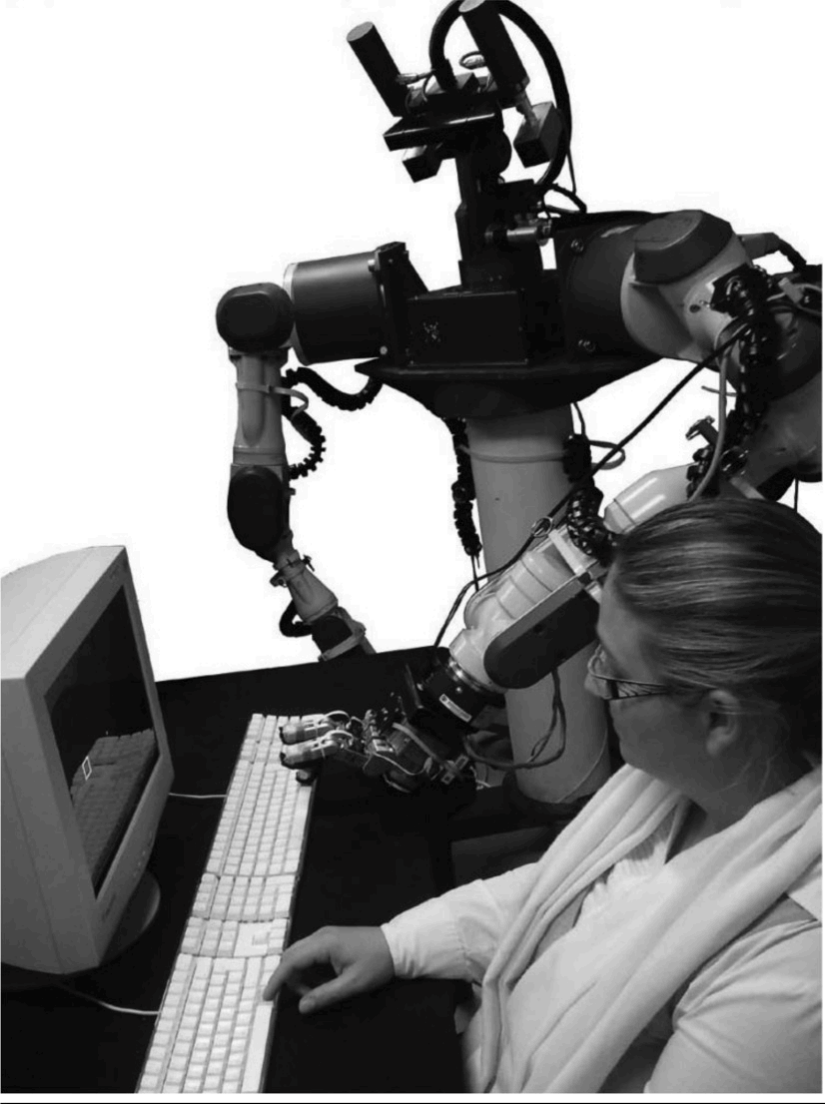
Experimental setting of the Social Simon task in the study of Stenzel et al. (2012).

Stenzel et al. (2012) also used a variant of the social Simon task (Sebanz et al., 2003) to investigate the human ability to represent actions that have been realized by a humanoid robot. In the study of Stenzel et al. (2012), the participants were sitting next to *Tombatossals*, a humanoid robot (with a torso, an anthropomorphic head, eyes-cameras, two arms, and a threefinger four-degrees-of-freedom left hand) described either as an intelligent and active agent or a passive machine acting in a deterministic way. The participants had to detect one type of target (e.g., a white square) that could appear on the left or the right side of a screen. The task of the robot was to detect another type of target (e.g., a white diamond) on the same screen. The experimental setting of the Social Simon experiment with a robot is presented in Figure 6. The authors analyzed the reaction times of the participants. Interestingly, the authors found a compatibility effect when the robot was introduced as a human-like robot who can actively act but not when the robot was introduced as a deterministic machine. Hence, the co-representation of other-generated actions can also occur during joint actions with automated artificial agents (and not only with human peers) provided that the robot is considered as a human-like partner. Probably, to envisage the others as similar to us is needed in order to map their actions into our own cognitive system and to compute a forward model.

Additionally, Glasauer et al. (2010) have shown that during hand-over interactions between a human being and a robotic arm, the predictability of the robotic arm motions for the human was strongly dependent on the automaton's motion laws and physical appearance (Glasauer et al., 2010). Indeed, when the robotic arm was handing on a cube to the human seated in front of it, his or her reaction times to grasp the object were faster when the robot assumed human-like kinematics in comparison with a trapezoidal joint velocity, meaning that individuals were able to better predict the observed human-like movement endpoints. Interestingly, the effect of the kinematic profile on the reaction times was modulated by the external appearance of the robot: the humanoid robot led its human partner to have faster reaction times than the industrial robot system. Moreover, the reaction times tended to be faster when the robotic arm had a typical robotic motion profile but a humanoid appearance than when the robotic arm had a human-like kinematic but an industrial appearance (Glasauer et al., 2010). Thus, low-level sensorimotor information processing can be overweighted by high-level cognitive adjustment mechanisms that take into account the physical appearance of the partner. Alternatively, it can be possible that the participants were differently engaged in the interaction depending on whether they were acting with a human-like partner or a clearly non-human-like partner so that reaction times were faster when the robot had a humanoid appearance. Indeed, Searle (1983) mentioned that to recognize the other as similar to oneself and also as a potential agent is a prerequisite to engaging in a collaborative activity. In any case, this result emphasizes the role of predictive mechanisms in human-robot interaction.

Research has also been focused on shared representation that occurs during action execution and observation. More specifically, Chaminade et al. (2005) were interested in the motor interference phenomenon that occurs when an individual has to perform an action while perceiving a different one. In such a case, the execution of the action is hindered because of the strong interdependence of the brain areas involved in action perception and in action execution. In their study, participants had to perform a rhythmic arm movement while observing another human or *DB*, a humanoid robot (with a torso, a head and two arms with hands devoid of fingers, each arm having seven degrees of freedom). In the *congruent* condition, the two agents had to perform rhythmic movements, each in a similar direction (e.g., two vertical motions, or two horizontal motions). In the *incongruent* condition, the two agents had to perform rhythmic movements, each in perpendicular directions (e.g., one vertical motion and one horizontal motion). The authors made the kinematic of the robot vary so that the robot kinematics could adopt biological or robotic motion laws. Motor interference was measured as the variability of the participants' kinematics. The results showed that the interference effect (more deviations in the participants' kinematics when the two agents performs different actions) occurred only when the robot had human-like motion, suggesting that individuals are less sensitive to artificial motions. During a joint action with a humanoid robot, like a synchrony task for example, we implicitly include our partner action into our own motor plan, if this observed action obeys biological laws. Artificial motion does not induce such shared representations, even if the robot has an anthropomorphic appearance.

#### Passive observation of machines

Many authors have argued that the action/observation matching predictive system could be activated during the observation of robot-generated actions (Press et al., 2005; Oberman et al., 2007; Kuz et al., 2015). In contrast, some PET studies found that the predictive system was not responsive to non-human-generated action, whatever its physical appearance or kinematic motion rules, and even when the goal of the action was understood by the observer (Perani et al., 2001; Tai et al., 2004).

##### Passive observation of traditional automated systems

A reaction time study by Press et al. (2005) demonstrated that the action/observation matching system was sensitive to the observation of robotic gestures (Press et al., 2005). Indeed, when individuals were primed by the picture of a compatible hand gesture (e.g., an opened posture), they were faster to perform this action then compared to when they were primed by an incompatible hand posture (e.g., a close posture), both for human and robotic hands. Nonetheless, this compatibility effect was stronger with the human primes.

At the cerebral level, Perani et al. (2001)'s showed that the predictive system was not activated during the observation of a non-humanized virtual hand (with 27 degrees of freedom) performing goal-directed actions, such as object grasping, even though the actions fitted a biological template (Perani et al., 2001). In their study, the hand stimulus used was not a robotic arm strictly speaking but even so, it was visually close to a non-humanized automated hand. However, it has also been brought to light that the predictive system could be activated during the observation of automaton-generated actions that did not follow biological motion laws (Gazzola et al., 2007). In fact, in their fMRI study, Gazzola and her colleagues demonstrated that the mirror brain areas involved in predictive mechanisms were activated while individuals were looking at videos of a non-humanoid robotic arm that was performing non-biological simple and complex object-directed actions. In this study, participants were shown videos of an agent's arm reaching and grasping familiar objects or just performing non-goal-directed movements without any object. The agent's arm could be either a human arm with biological motion laws or a non-humanoid robotic arm with robotic motion laws. Thus, while the goal of the actions was the same, the way this goal was achieved clearly differed according to the effector's motion properties. The predictive system was similarly solicited during the human and the robotic actions suggesting that the kinematic properties of the action did not matter. Indeed, it has been hypothesized that when an observer is familiar with an action goal, deviations (from our own motor system) in kinematics can be passed over and it is still possible to elicit the observer's predictive system. Following this line of thought, the congruence between a represented goal and an observed goal is enough to trigger the predictive system activation (Gazzola et al., 2007). On another side, a TMS study by Craighero et al. (2016) showed the involvement of the motor system during the passive observation of simplistic hand action representations (using a point light display), both when the kinematics were biological and non-biological, with no difference between the two conditions, and even though the stimuli were not perceived as hands (Craighero et al., 2016).

##### Passive observation of humanized automated systems

Behavioral studies indicate that individual's predictive abilities increase when they observe automata that are implemented with human-like motion laws (Sciutti et al., 2014). For example, Sciutti et al. (2014) investigated individual's ability to infer an object weight from an observed robotic-generated action directed toward the object. In their study, participants had to watch movies where either a human or a humanoid robot *iCub* (with a torso, an anthropomorphic face and two arms with five fingers, each hand having nine degrees of freedom) was manipulating an object. The object was identical in all movies but its weight could vary from 100 to 400 g. The authors manipulated the kinematics of the robot, replicating a human-like biological correlation between action kinematics and object weigh (see “proportional condition” in the paper). After each movie, the participants had to judge the perceived weight of the object, with a rating scale from 1 to 9 corresponding to weights from 50 to 450 g. The authors showed that participants could infer the mass of the object from the robot's action as accurately as from the observation of the human lifting. This supports that observers are sensitive to biological motion, and by simulating the observed action that is part of their own motor repertoire (through the forward model), they can infer an object property that is not accessible through vision, with the help of their motor expertise (through the inverse model). This is minimized when the robot motion is not human-like, even if it has a humanoid physical appearance.

At the cerebral level, the issue of whether or not biological motion can activate the observer's predictive mechanisms remains controversial. Using fMRI, Kuz et al. (2015) asked participants to watch videos showing the arm of an agent displacing a cylinder. The physical appearance of the arm was either human or industrial robotic and the motion kinematic was either human-like or robotic-like. The results revealed that brain areas involved in predictive mechanisms were activated during the observation of the human-like and the robotic-like actions, with a stronger activation for the former (Kuz et al., 2015). Likewise, using electroencephalographic recording on volunteers who were watching a video showing a humanoid robotic arm grasping a ball with human-like motion properties or performing the very same action in the absence of the object, it has been shown that the observer's predictive system was elicited in both cases (Oberman et al., 2007). Both studies involved actions performed by a robot with biological features, which could explain why the predictive system was strongly engaged. Indeed, Ulloa and Pineda (2007) showed that the action biological kinematic content *per se* was enough to solicit the observer's predictive system (Ulloa and Pineda, 2007).

On the contrary, it has been shown that the observation of a non-biological reach-to-grasp action executed by a humanoid robotic arm did not elicit the predictive system (Tai et al., 2004). However, Gazzola et al. (2007) have tried to understand why Tai et al. (2004) failed to find predictive system activation during robotic action observation. To do so, they scanned participants who were watching a robot perform either 5 different actions within a block (as in their previous described experiment) or 5 times the same action within a block (as in Tai et al. study). In the first case, the author found a significant activation of predictive system whereas no such effect occurred in the latter case. Hence, the authors proposed that robot-generated actions could activate the observer's predictive system as soon as different actions are shown in the same block in order to avoid habituation effect (Gazzola et al., 2007).

To sum up, even if there is no clear consensus, most of the neuroimaging studies seem to report the activation of predictive mechanisms during the observation of actions that have been generated both by humanized automated systems and by traditional automated systems (see Table 1 for a summary of the neuroimaging results). Nevertheless, behavioral data emphasizes the point that the more the machine is humanized (through its physical appearance or its kinematics), the more the human-machine interactions are improved.

**Table 1:** Comparison of neurophysiological studies investigating the link between robotic generated action observation and predictive system activation.

**Study**	**Robotic arm used**	**Robotic arm with a human-like appearance**	**Robotic arm with human-like motion properties**	**Method used**	**Observer's predictive system activation**
Gazzola et al., 2007	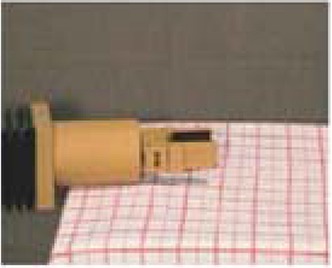			fMRI	^*^
Kuz et al., 2015	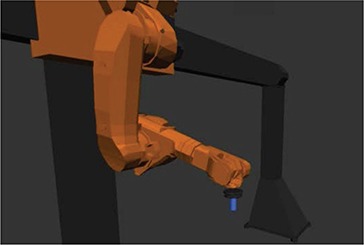		(^*^)	fMRI	^*^
Oberman et al., 2007	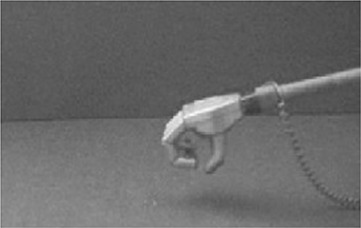	^*^	^*^	EEG	^*^
Perani et al., 2001	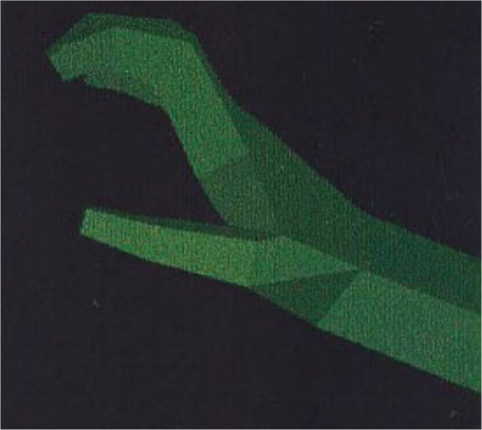	^*^	^*^	PET	
Tai et al., 2004		^*^		PET	(^*^)

“^*^”means that the robotic arm used in the mentioned study has the characteristics described at the top of the column (in the label).

### Predictive systems for the sense of we-agency with machines

Numerous studies related to agency in dyads tend to highlight a clear distinction between the sense of agency felt by an agent when he is interacting with another human vs. with a simplistic machine (Wohlschläger et al., 2003a,b; Obhi and Hall, 2011b; Poonian and Cunnington, 2013; Sahaï et al., 2017).

#### The sense of agency during joint actions with machines

##### The sense of agency during joint actions with traditional automated systems

In Obhi and Hall (2011b)'s study, the participants had to tap a touchpad at the time of their choice, which triggered a tone after a certain delay. The participants were either paired with a human partner or with a computer, separated from them by a curtain. They thought that their partner could also trigger the tone but in fact, it was always the naïve participant who triggered the sensory effect. The participants had to judge the onset time of their own action or the tone with the help of a clock. Participants' beliefs about the initiator of the action always fitted the false given feedback. Interestingly, during the joint actions with the computer, the participants did not demonstrate IB when they were convinced that the tone was machine-generated, and most intriguing, when they were convinced that they were responsible for the tone. That is, regardless of participants' beliefs no experience of agency took place despite the fact that it was indeed the participant who caused the sensory effect. To explain these results, the authors argued that the automatic “we identity” formation may not occur when the partner is not a human being (Obhi and Hall, 2011b). Also, one must take into consideration the cue integration framework which suggests that the sense of self-agency is built by the dynamic integration of internal and external cues (Moore and Fletcher, 2012). Indeed, these authors proposed that both internal motoric signals and external situational signals contribute to the sense of self-agency via an integrative processing in which each cue is weighted by its reliability, with a higher intrinsic weighting for the internal cues by default. However, under certain circumstances (e.g., when the motoric information is weak or absent and when the external cues are subsequently presented), internal cues can be bypassed so that the sense of self-agency becomes mainly context-dependent (Moore et al., 2009). In Obhi and Hall's (2011b) study, a false feedback about the presumed initiator of the tone was given to the participant after each trial. This could have reinforced the situational cues weighting, explaining why the explicit judgments of self-agency of the participants always matched the given feedback despite the fact that the participants always performed the action that triggered the tone. However, at the implicit level, the participants did not manifest IB even when they were convinced (with the feedbacks) that they had effectively triggered the sensory consequence. This suggests that during joint actions with a machine, high-level meta-cognitive knowledge about the nature of the partnership can disrupt the sense of self-agency if the partner involved in the shared task is not an intentional agent.

In addition, Sahaï et al. (2017) investigated the sense of we-agency during interactions with a computer. They made participants estimate temporal intervals between the computer target detections and a tone that was triggered 400, 900, or 1,400 ms after the detection. These estimations had to be performed either during the passive observation of the computer (*computer observation* condition) or when the participants had to perform a complementary target detection task with the computer (*human-computer joint go-nogo* condition). The temporal estimations served as an implicit measure of the participants' sense of agency over the action performed by the computer. The authors found no differences in the participants' estimations between the *computer observation* condition and *human-computer joint go-nogo* condition. Indeed, no temporal attraction occurred in the joint action condition, supporting that being involved in a common task with a traditional automaton, such as a computer does not create a sense of shared agency in the human cognitive system. This is to relate to the absence of Social Simon effect during joint actions with a computer (Sahaï et al., 2017). An interesting perspective could be to implement an interface with a virtual avatar in order to see if the sense of joint agency could be enhanced. Indeed, it can be possible that interacting with a human-like agent could stimulate the co-representation of the action performed by the computer, reinforcing the sense of we-agency.

##### The sense of agency during joint actions with humanized automated systems

In an interesting study by Caspar et al. (2016), the participants were wearing a glove with sensors on their right hand (hidden from vision) so that they could control a human-like robotic right hand that was placed in full view in front of them. The participants learnt during an association phase a given keypress (H or F key) would trigger a specific tone (a 400 or 600 Hz tone). In the *robot homologous-tone congruent* condition, the participant had to press either the H or F key whenever he or she wanted and the robotic hand immediately did the same action. The robot's key press triggered a tone which was congruent with the tone learnt in the association phase. In the *robot homologous-tone incongruent* condition, the participant had to press one of the two keys whenever he or she wanted and the robotic hand immediately did the same action. However, the robot's key press triggered a tone which was incongruent with the tone learnt in the association phase. In the *robot non-homologous-tone congruent* condition, the participant had to press either the H or F key whenever he or she wanted but the robotic hand immediately did the opposite action. The robot's key press triggered a tone which was congruent with the tone learnt in the association phase. In the *robot non-homologous-tone incongruent* condition, the participant had to press one of the two keys whenever he or she wanted but the robotic hand immediately did the opposite action. In addition, the robot's key press triggered a tone which was incongruent with the tone learnt in the association phase. The participant had to estimate the temporal interval between the key presses and the following tones. During the experiment, the electroencephalographic activity of the participant was recorded. The authors found that when the robot action was homologous to the participant action, the participants made more IB when the tone was congruent compared to when it was incongruent. This effect of congruency did not exist anymore when the robot action was incongruent with the participant action. This indicates that the association between a given action and its sensory consequence is not the only thing that matters for the sense of agency. In addition, the means used to realize the desired outcome is important. The authors proposed that the sense of agency was mainly informed by an online tracking control process that can predict the intermediate steps along the causal chain. When a disruption occurs, the sense of control over the other-generated action is hence reduced. In addition, the authors have found a N1 auditory component amplitude reduction for congruent tone compared to incongruent tone only when the robot action was homologous. Such a sensory attenuation can be considered as a marker of the processing of self-generated effects (Blakemore et al., 2000; Poonian et al., 2015).

#### The sense of agency during passive observation of machines

Similarly, a clear distinction between the sense of agency felt by an individual after observing another human vs. a machine has been made by several authors (Poonian and Cunnington, 2013, experiment 2; Wohlschläger et al., 2003a,b). As no shared goal is engaged, the sense of agency may be mediated by the predictive system activations. However, failures in predictive mechanisms during the observation of machines may be at the source of the difficulties experienced in having a sense of agency during machine-generated actions (Wohlschläger et al., 2003a,b).

In Wohlschläger et al. (2003b)'s study, participants were placed in front of a lever that triggered a tone 250 ms after being pushed on. There was a *machine-action* condition in which participants looked at the lever move automatically. Participants had to estimate the onset time of the lever press. The perceived onset time of the action in the *machine-action* condition was perceived earlier than the onset of self-generated and other-human-generated actions. In another experiment, Wohlschläger et al. (2003a) replicated the paradigm of the Wohlschläger et al. (2003b)'s study except that this time, the participants, the experimenter and the lever were wearing a rubber glove (Wohlschläger et al., 2003a). The participants' task was to estimate the onset time of the lever press. Results in *self-action* and *other-action* conditions fitted those of Wohlschläger et al. (2003b)'s experiment. However, for the *machine-action* condition, the authors found a significant difference compared to the former experiment. Indeed, when the lever was wearing the rubber glove, participants judged the onset of the action closer to the tone than when the lever had a usual appearance, but it was still distinctive from the perceived onset of self and other generated actions. Thus, when the actor was a machine, individuals did not sense agency even though they tended to when the machine had humanoid physical features. In another study (Poonian and Cunnington, 2013), the participants had to observe a video showing either a human or an automated system button press that triggered a tone after a certain temporal delay that they had to estimate. The participants expressed less IB when the sensory effect was triggered by the automated system compared to the biological agent. In addition, no perceptual attenuation occurred when perceptual effects were triggered by an observed machine (Poonian et al., 2015).

In sum, joint action with a machine and passive observation of a machine can create a cognitive gap compared to human interactions (Norman and Draper, 1986; Limerick et al., 2014). This problem might stem from the opacity of traditional automated system that restrains human ability to predict the system's intentions. Thus, two non-exclusive hypotheses are opened to us. According to the first hypothesis, the sense of we-agency relies on a low-level motor prediction system. During joint actions with traditional automated systems, the predictive system fails to be engaged because humans cannot simulate the machine motor schemes using their own cognitive system (e.g., a button or lever automatic depression). According to the second hypothesis, the high level belief according to which we are interacting with an automated system (e.g., in Obhi and Hall, 2011b study) is enough to inhibit the sense of we-agency. Regrettably, no study to date has investigated the relationship between the potential action/observation matching system activation elicited during interaction with human-like automata and one observer's sense of agency.

## Conclusion

Predictive mechanisms are involved in action control and also in the sense of agency (Blakemore et al., 2002; Synofzik et al., 2008). Empirical data tend to show that predictions allow better coordination with human peers in joint actions and support action understanding of observed agents (Manera et al., 2011). Predictive mechanisms are also involved in the sense of agency formation during the observation of actions (Wohlschläger et al., 2003a). However, humans experience difficulties when they have to collaborate with traditional machines (Mann et al., 2007; Poonian et al., 2015; Sahaï et al., 2017). IB does not occur when humans are observing a traditional automaton acting or when they act in partnership with this sort of automaton (Wohlschläger et al., 2003a,b; Obhi and Hall, 2011b; Poonian and Cunnington, 2013; Sahaï et al., 2017). This has prompted some authors to attempt to give the machine a more human-like appearance in order to enhance human's sense of agency during interactions with robots (Wohlschläger et al., 2003b). Consistently, it appears that humanized artificial systems can help moderate participants' difficulties in coordinating their actions with them and experiencing a sense of agency when they interact with machines (Wohlschläger et al., 2003b; Glasauer et al., 2010). Moreover, the implementation of biological motion laws in anthropomorphic robot allows a better implicit understanding of these actions thanks to observer's action/observation matching system, with the help of his or her own motor experience. Indeed, considering that operators interpret the intentions and the action outcomes of a system with their own “cognitive toolkit,” to implement human-like motions in robot can make it easier for humans to predict the machine actions. In addition to make automated systems more predictable (i.e., to optimize intention understanding from early action observation), maximizing action legibility (i.e., to facilitate action reading from kinematics) might be another requirement for machine-generated action better understanding (Dragan et al., 2013). Finally, even though research in social robotic keeps trying to understand how to optimize the interactions between social robots and humans, investigations about the sense of agency during these joint actions are still missing. Optimizing human robot interaction is a crucial issue for the design of future technological systems given that humans will be increasingly involved in tasks where they need to interact with highly automated environment. The science of agency can help a better comprehension about how individuals can have a sense of control over these automata.

## Author contribution

All authors listed have made a substantial, direct and intellectual contribution to the work, and approved it for publication.

### Conflict of interest statement

The authors declare that the research was conducted in the absence of any commercial or financial relationships that could be construed as a potential conflict of interest.
