# Corrigendum: Predictive Mechanisms Are Not Involved the Same Way during Human-Human vs. Human-Machine Interactions: A Review

**DOI:** 10.3389/fnbot.2018.00008

**Published:** 2018-03-07

**Authors:** Aïsha Sahaï, Elisabeth Pacherie, Ouriel Grynszpan, Bruno Berberian

**Affiliations:** ^1^Département d’Etudes Cognitives, ENS, EHESS, Centre National de la Recherche Scientifique, Institut Jean-Nicod, PSL Research University, Paris, France; ^2^ONERA, The French Aerospace Laboratory, Département Traitement de l’Information et Systèmes, Salon-de-Provence, France; ^3^Institut des Systèmes Intelligents et de Robotique, Université Pierre et Marie Curie, Paris, France

**Keywords:** prediction, sense of agency, comparator model, joint action, HMI

**Incorrect Reference**

In the original article, the reference for **Caspar,** et al.**, 2016** was incorrectly written in the reference list as **Caspar, E. A., Christensen, J. F., Cleeremans, A., and Haggard, P. (2016). Coercion changes the sense of agency in the human brain. *Curr. Biol*. 26, 585–592. doi:10.1016/j.cub.2015.12.067**

It should be **Caspar, E. A., Desantis, A., Dienes, Z., Cleeremans, A., and Haggard, P. (2016). The sense of agency as tracking control. *PLoS ONE* 11(10):e0163892. doi:10.1371/journal.pone.0163892**

The authors apologize for this error and state that this does not change the scientific conclusions of the article in any way.

The original article has been updated.

## Conflict of Interest Statement

The authors declare that the research was conducted in the absence of any commercial or financial relationships that could be construed as a potential conflict of interest.

